# Effect of vacuum cooling on stability of macro‐porous sausage during refrigerated storage—Vacuum‐cooled sausage has a longer shelf life

**DOI:** 10.1002/fsn3.1435

**Published:** 2020-03-28

**Authors:** Xiao‐yan Song, Zuo Song, Baolin Liu, Zhi‐yu Guo, Yuchen Luan

**Affiliations:** ^1^ Institute of Cryobiology and Food Freezing University of Shanghai for Science and Technology Shanghai China

**Keywords:** macro‐porous, sausage, storage, vacuum cooling

## Abstract

In this study, two types of cooling methods (vacuum cooling and air cooling) were used to cool cooked macro‐porous sausage. Alterations in the microbiological conditions, pH, instrumental color (L*, a*, and b*), total volatile nitrogenous bases (TVB‐N), lipid oxidation (TBARS), water activity (aW), moisture content, and texture indicators were evaluated to determine sausages' quality changes during storage under refrigeration for up to 10 days. In general, the shelf life of sausages chilled by vacuum cooling (8 days) was similar to that of sausages cooled by air cooling (9 days). For pH, no significant difference (*p* > .05) was obtained between two cooling methods. However, vacuum‐cooled sausages have lower L* value (*p* < .05), lower moisture content, and water activity compared with the air‐cooled sausages. However, sausages cooled by vacuum cooling showed a sharp increase in TBARS and TVB‐N values but maintained texture characteristics for a longer time compared with air‐cooled sausages. Although the results indicated that the quality of sausages treated by those two methods remarkably decreased after 7 days, characteristics of sausages cooled by vacuum cooling are better within accepted standards compared with air‐cooled sausages. In conclusion, vacuum cooling can be a feasible cooling method with great potential to be used in cooked macro‐porous sausages to maintain the quality and may provide reference experiences for the food with similar structure.

## Graphical abstract

Two types of cooling methods (vacuum cooling and air cooling) were used to cool cooked macro‐porous sausages, which will store almost 10 days at 4°C. Storage stability is primarily assessed by physical, chemical, and microbial parameters.

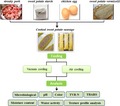

## 1 INTRODUCTION

Drying, freezing, cooling, and modified atmosphere packing have been used to improve the shelf life of food in the recent years. Baldini et al. (2000) proposed that better surface flora can be obtained with dry conditions. Shen et al. (2010) also showed that the quality of Whangkeumbae pear frozen at −1~0°C was better than that of pear frozen at 1.5°C. Kumar, Kumar, and Murthy (2008) reported that the precooling process after harvest can prevent food spoilage by using air, water, or both cooling methods. Moreover, studies indicated that packing conditions can also significantly ameliorate the shelf life of products (Fernández‐Fernández, Vázquez‐Odériz, & Romero‐Rodríguez, 2002; Zanardi, Dorigoni, Badiani, & Chizzolini, 2002). However, all these methods have some disadvantages in practical use. For example, Eerola, Sagués, Lilleberg, and Aalto (1997) noticed that dry sausages after the fermentation could also be contaminated by amine‐producing bacteria. Schmidt, Silva, Zanoelo, and Laurindo (2018) observed that air‐cooled samples have slower cooling rates and more counts of mesophiles and psychrophiles than that of vacuum‐cooled samples. Therefore, the application of vacuum cooling is currently being researched to extend the shelf life of food. Compared to other techniques, vacuum cooling has rapid cooling rates, which can minimize microorganisms' growth. Vacuum cooling is completely different from other conventional cooling methods. It is performed by removing heat rapidly through evaporation of water from the product's surface and pores. This technique is now considered as one of the most useful methods for food preservation (Santana et al., 2018).

Since vacuum cooling can prolong the shelf life and enhance microbiological safety of food, this technique has been widely used for precooling fruit, vegetables, meat, meat products, bakery products, and ready meals (Mcdonald, Sun, & Kenny, 2000). In addition, the vacuum cooling of cooked meat and the comparison with other cooling methods has been extensively reported. Ozturk, Ozturk, and Koçar (2017) inspected the microbial growth rate of meatballs cooled by vacuum cooling and conventional cooling, respectively. Feng, Sun, Martín, and Zhang (2013) observed that cooked sausages operated by immersion vacuum cooling in cold water can achieve a longer shelf life than immersion vacuum cooling in hot water and commercial cooling. In the paper from Qiao, Zhang, and Ren (2012), vacuum cooling prolonged the shelf life of emulsion‐type sausage. However, most of the available studies have focused on evaluating the benefits of vacuum cooling of cooked compact meat rather than macro‐porous food (Drummond & Sun, 2008; Feng et al., 2013; Song, Guo, Liu, & Jaganathan, 2018).

Macro‐porous foods, such as sweet potato sausage, which is one of the important kinds of processed meat products, are very popular in China. The basic ingredients of sweet potato sausage are streaky pork, sweet potato vermicelli, sweet potato starch, corn oil, chicken eggs, and salt. Since meat belongs to perishable food products on the market (Cannarsi et al., 2005), the shelf life of sweet potato sausage is less than 24 hr without timely refrigeration after cooking. Therefore, after manufacturing, the sausage is subjected to a preservation treatment with the aim of increasing its shelf life and preventing from microbial spoilage.

The objective of this study was to investigate the effect of vacuum cooling on macro‐porous food and provide reference experiences for the similar food structure. In this study, the experiments were carried out on the vacuum cooling and the air cooling for cooling sweet potato sausage. Furthermore, effects of different cooling methods on the microbial, physicochemical, and textural properties of the cooked sausages during stored 4°C were also explored.

## 2 MATERIALS AND METHODS

### 2.1 Sample preparation

#### 2.1.1 Sausage stuffing processing

Sausages were prepared in laboratory with 100 g streaky pork plus 45 g water, 100 g sweet potato vermicelli, and 150 g sweet potato starch added to the following common ingredients in order: 10 g salt, one chicken egg white and 7 g corn oil and mixed. All ingredients were bought from LOTUS supermarket (Shanghai, China).

#### 2.1.2 Sausage coating processing

The sausage coating was made of chicken eggs instead of natural casing. Four whole chicken eggs and four chicken egg yolks were put into a bowl and stirred well. A little sweet potato starch solution was added to the mixture (the proportion of water and sweet potato starch should be 1:1) to make the omelet stronger and resist breaking. About 55 g of this mixture was poured into a pan (CS‐M2, Catering Value) heated using induction cooker (SDHCB9E88‐210, SUPOR). The concentrate was cooked for 2–3 min.

#### 2.1.3 Sausage processing

Put the filling on the cooked sausage coating and then rolled the sausage into cylindrical shape with the aid of thin plastic film.

### 2.2 Cooking and cooling treatments

All samples were placed in a steamer (SZZ26B5, SUPOR) and steamed for 30 min with 100°C. For vacuum cooling, cooked sausages were transferred immediately into vacuum chamber and cooled the core temperature to below 4°C. Then, the samples were vacuum packaged (vapor permeability is 328.55·10^–6^ cm^3^ m^−2^ day^−1^ Pa^−1^, moisture permeability is 15 g m^−2^ day^−1^) and stored at 4°C. The vacuum cooling treatment was implemented by a vacuum cooler (Song, Liu, & Jaganathan, 2016). For air cooling, cooked sausages were vacuum packaged and cooled at room temperature by a fan (KYT‐3001a, Gree) and then stored at 4°C. Temperature changes in the center of sausages during these two cooling procedures were monitored by thermo‐couples (T‐types, OMEGA Engineering).

Before the experiment, all the equipment was sterilized by 75% alcohol and ultraviolet lamp. All of samples were stored at 4°C (70–80 RH) for 10 days. Each treatment of packaged sausage was randomly taken for analysis every day until they were inedible. The shelf life of sausages was evaluated by the range of alterations in the microbiological conditions, pH, instrumental color, total volatile nitrogenous bases (TVB‐N), lipid oxidation (TBARS), water activity (aW), moisture content, and texture indicators.

### 2.3 Microbiological and pH analysis

About 25 g sweet potato sausage was homogenized using 225 ml physiological saline (0.85% NaCl) and serially diluted with the same diluent. Then, 0.1 ml of the diluted sample was spread on plate count agar. Plates were inverted and incubated at 36°C for 48 hr. The microbiological method is according to the procedure of National food safety standard‐ food microbiological examination: Aerobic plate count (GB 4789.2‐2010). pH was measured by blending 5 g of product with 45 ml of distilled water for 2 min and measured with a digital pH meter (PHS‐25, LEICI) according to the document of GB 5009.237‐2016.

### 2.4 Color analyses

Color measurements of samples were measured according to the methods reported by Feng et al. (2016). All measurements were taken in triplicates.

### 2.5 Total volatile bases‐nitrogen and thiobarbituric acid reactive substances

Total volatile bases‐nitrogen (TVB‐N) was determined by acetyl‐acetone‐formaldehyde spectrophotometry described by Zhang and Li (2001). Thiobarbituric acid reactive substances (TBARS) were measured following the method proposed by Ke, Cervantes, and Robles‐Martinez (1984). The amounts of TBARS were expressed in milligrams of malondialdehyde (MDA) per 100 g of sample. Both of the reactions' absorbency was recorded at *λ* = 412 nm and *λ* = 532 nm using a spectrophotometer (752N, INESA), respectively.

### 2.6 Moisture content and water activity measurements

Moisture content of the sausage during storage was estimated by measuring its fresh and dry weight obtained before and after drying 5–8 g samples. The samples inside a crucible were dried in an oven at 105°C for 4 hr. The final moisture content is expressed as percentage of moisture with a precision of 0.01.

The water activity (aw) of samples was measured using AquaLab 4TE (Decagon). After machine running for 30 min, the chopped sausages covered the bottom of sample cup to test water activity.

### 2.7 Instrumental texture profile analysis

The instrumental texture profile analysis (TPA) of sausage samples was performed using the double bite‐size method by a texture analyzer TA.XTplus (SMS). Eight cubes of sweet potato sausage (1 × 1 × 1 cm) were compressed twice with a cylindrical probe of 5 cm diameter, at 5 mm/s speed and the level of compression was 50% of the thickness of the sample. The textural properties of sausage were expressed as hardness, springiness, cohesiveness, resilience, and chewiness.

### 2.8 Statistical analysis

All the measurements were taken in triplicate for each group at each measurement period, and the mean values ± standard deviation (*SD*) were calculated for the analysis. Experimental data were tested by analysis of variance (ANOVA), and means separation was achieved using Duncan test at 95% confidence, using SPSS (IBM, V22.0).

## 3 RESULTS AND DISCUSSION

### 3.1 Microbiological and pH analysis

**Figure 1:**
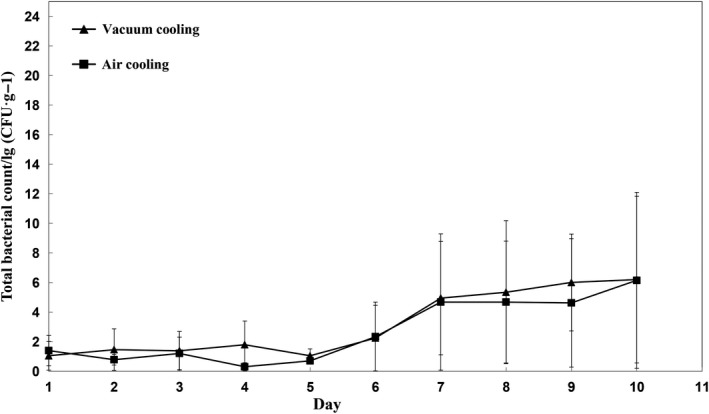
Effects of cooling methods and storage time on total bacterial count of sweet potato sausage stored at 4°C

The total plate counts of all samples increased during refrigerated storage (Figure 1). Both cooling method and storage time significantly affected the microbial numbers especially after 6 days of storage (*p* < .05). The interaction between the cooling methods and storage time has significant effect (*p *< .05) on the microbial numbers at the same time. Although most of bacteria were killed by cooking, some microorganisms can still survive. Feng et al. (2013) noted that the best temperature affecting microorganism growth was 63~5°C. This means the cooling rates followed by cooking procedures decided the microenvironments, which are constantly changing in food products. The faster the cooling rate is, the less likely the microorganisms generate. Previous study has indeed demonstrated that cooling rates influenced the growth of microbes (Mcdonald et al., 2000). For safety, a minimum temperature‐time treatment should be achieved during cooling after cooking (Burfoot, Self, Hudson, Wilkins, & James, 1990). Table 1 shows that the time needed for vacuum cooling from about 80°C to 4°C is significantly shorter than air cooling (*p* < .05), which further shows that vacuum cooling is an efficient cooling method compared with air cooling. As for air cooling, the poor convection between sausage and air could be a reasonable explanation for longer cooling time.

**Table 1:** Cooling rates of sausages chilled by different methods from 80°C to 30‐4°C

Cooling methods	Cooling rates (℃/min; mean ± *SD*)
Air cooling	0.804 ± 0.004^A^
Vacuum cooling	7.866 ± 1.462^B^

Note

The same column with different superscript letters are significantly different for different cooling methods (*p* < .05).

**Figure 2:**
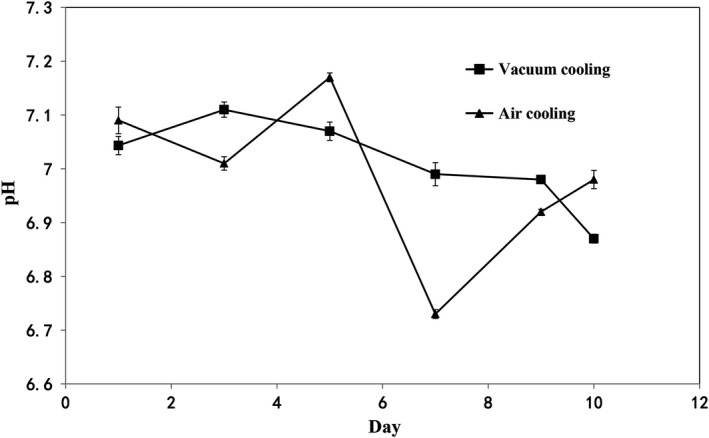
Effects of cooling methods and storage time on pH of sweet potato sausage stored at 4°C

Although cooling rate of vacuum cooling is faster than air cooling, the microbial flora of samples cooled by two cooling methods increased slowly before 5th day and then increased sharply from 6th day. This increased tendency could be explained by stress imposed during cooking, which could have affected microorganisms' ability to cope with the environment. In any food environment, certain microbial species will survive and become dominant. According to the results shown in Figure 2, pH values gradually decreased during the refrigerated storage. No significant differences were found between the different cooling methods in terms of pH (*p* > .05). The initial pH of sausages was over 7.0, and then, pH values had a progressive decrease until dropped below 7.0. However, the vacuum cooling maintained the sausages' pH values roughly equally until the 9th day of storage. The change of pH during storage with different cooling methods was also reported by Feng et al. (2013). The rise of pH at the beginning can be attributed to nitrogen compounds, and however, the effect of microbial growth, especially lactic acid bacteria, was greater than that of nitrogen compounds, which results in the reduction of pH during the later refrigerated storage. This phenomenon could probably result from the *Lactobacilli*, which is frequently present in fresh meat (Salazar, García, & Selgas, 2009) and gradually became the dominant flora (Feng et al., 2013) during the storage.

In this work, the shelf life of the sausages chilled by vacuum cooling was 8 days, which is shorter than that of sausages cooled by air cooling (9 days). And Burfoot et al. (1990) once observed that convective air cooling, water immersion cooling and pressure/vacuum cooling may not affect the bacterial condition for large meat joints. Such difference in this work could be explained by macro‐porosity of the cooked sweet potato sausage. The macro‐porous sausage created suitable environments that encourage the growth of bacteria.

### 3.2 Color measurements

The results of lightness (L*), redness (a*), and yellowness (b*) color values of the sweet potato sausage treated by different cooling methods are presented in Tables 2, 3, 4. In terms of L* value of sausage, significant differences exist between vacuum cooling and air cooling (*p* < .05). Lightness increased significantly (*p* < .05) with storage time, and interaction between the cooling methods and storage time was also significant (*p < *.05) effect of the lightness. On the one hand, the upward tendency is probably related to the presence of starch, which retain moisture in processed meat (Zamudioflores et al., 2015). And Feng et al. (2013) found that lightness increased with increasing moisture content. On the other hand, the lightness increased as pH decreased during storage at cold room. Allen, Russell, and Fletcher (1997) observed that dark‐colored meat has lower water content and higher pH than light‐colored meat. Briskey and Kauffman (1971) found that when meat with high pH value over isoelectric point, water molecules are bound tightly, causing more light to be absorbed by muscle, which show darker in lightness. Besides, Allen et al. (1997) found that dark‐colored meat has shorter shelf life than light‐colored meat. In this study, vacuum‐cooled sausages were darker than air‐cooled sausages, indicating that sausages cooled by vacuum cooling have a shorter shelf life than sausages cooled by air cooling. However, no significant differences (*p > *.05) were found between core lightness and surface lightness, which is not coincident with the conclusion made by Feng et al. (2013), who showed that core sausage color was darker than surface color because fat stay in the casing.

**Table 2:** Effects of cooling methods and storage time on lightness (L*) of sweet potato sausage stored at 4℃

	Core of sausage							Surface of sausage					
	Day1	Day3	Day5	Day7	Day9	Day10		Day1	Day3	Day5	Day7	Day9	Day10
VC	61.95 ± 0.45^Aa^	62.84 ± 0.46^Abc^	63.05 ± 0.33^Acd^	63.95 ± 0.92^Ad^	63.59 ± 0.25^Acd^	63.64 ± 0.38^Ad^	VC	62.39 ± 0.39^Aa^	63.16 ± 0.54^Ab^	63.53 ± 0.40^Abc^	63.65 ± 0.43^Abc^	63.34 ± 0.40^Ab^	63.89 ± 0.51^Ac^
AC	64.18 ± 0.50^Bb^	65.10 ± 0.73^Bc^	63.23 ± 0.31^Aa^	65.41 ± 0.61^Bc^	65.60 ± 0.63^Bc^	65.71 ± 0.85^Bc^	AC	64.34 ± 0.83^Ba^	65.26 ± 0.40^Bc^	63.81 ± 0.52^Aa^	65.03 ± 0.50^Bb^	65.19 ± 0.56^Bb^	66.12 ± 0.48^Bc^

Note

The same row with different superscript lowercase letters are significantly different for different storage time (*p* < .05) and different superscript uppercase letters are significantly different for different cooling methods (*p* < .05).

**Table 3:** Effects of cooling methods and storage time on redness (a*) of sweet potato sausage stored at 4°C

	Core of sausage							Surface of sausage					
	Day1	Day3	Day5	Day7	Day9	Day10		Day1	Day3	Day5	Day7	Day9	Day10
VC	0.01 ± 0.40^Aa^	−0.42 ± 0.10^Abc^	0.70 ± 0.10^Acd^	−0.14 ± 0.33^Acd^	−1.26 ± 0.55^Ae^	−0.93 ± 0.29^Ade^	VC	−0.23 ± 0.22^Aa^	−0.44 ± 0.16^Aab^	−0.71 ± 0.12^Abc^	−0.65 ± 0.26^Abc^	−1.41 ± 0.38^Ad^	−0.88 ± 0.38^Ac^
AC	−0.32 ± 0.37^Aa^	−0.06 ± 0.69^Aa^	−0.24 ± 0.20^Ba^	−0.53 ± 0.56^Aa^	−0.04 ± 0.87^Ba^	−0.37 ± 0.57^Ba^	AC	−0.18 ± 0.44^Aa^	−0.42 ± 0.36^Aab^	−0.28 ± 0.10^Ba^	−0.87 ± 0.57^Abc^	0.09 ± 0.64^Ba^	−1.14 ± 0.73^Ac^

Note

The same row with different superscript lowercase letters are significantly different for different storage time (*p* < .05) and different uppercase superscript letters are significantly different for different cooling methods (*p* < .05).

**Table 4:** Effects of cooling methods and storage time on yellowness (b*) of sweet potato sausage stored at 4°C

	Core of sausage							Surface of sausage					
	Day1	Day3	Day5	Day7	Day9	Day10		Day1	Day3	Day5	Day7	Day9	Day10
VC	3.28 ± 0.60^Aa^	3.47 ± 0.45^Aa^	3.41 ± 0.23^Aa^	6.74 ± 0.99^Ac^	6.61 ± 0.64^Ac^	5.91 ± 0.39^Ab^	VC	3.26 ± 0.39^Aa^	3.65 ± 0.35^Aab^	3.80 ± 0.43^Ab^	6.48 ± 0.24^Ac^	6.55 ± 0.30^Ac^	6.64 ± 0.65^Ac^
AC	3.04 ± 0.30^Ab^	3.20 ± 0.38^Ab^	2.33 ± 0.27^Ba^	5.88 ± 0.42^Ad^	4.97 ± 0.51^Bc^	5.85 ± 0.21^Bd^	AC	3.32 ± 0.34^Ab^	3.05 ± 0.56^Bb^	2.55 ± 0.16^Ba^	5.68 ± 0.60^Bc^	5.44 ± 0.24^Bc^	5.83 ± 0.43^Bc^

Note

The same row with different superscript lowercase letters are significantly different for different storage time (*p* < .05) and different superscript uppercase letters are significantly different for different cooling methods (*p* < .05).

Table 3 shows the effect of cooling methods and storage time on redness (a*) of cooked sweet potato sausage stored at 4°C. Significant changes were found between redness of vacuum cooling and air cooling (*p* < .05), and sausage chilled by two cooling methods appeared significant (*p *< .05) decreased redness as the storage time increased. This finding may probably be due to the influence of oxidation from light and oxidation. Besides, sausage with high pH was redder than sausage with low pH was found by Yang and Chen (1993). This can explain why sausages' redness decreased with storage time. Allen et al. (1997) observed that as the pH decreased, lightness and yellowness values increased, while the redness values decreased. As can be seen in Table 4, the storage time has significant effect on yellowness values that increased gradually during the whole period of storage (*p* < .05). And the results shown that sausage treated by vacuum cooling were yellower than that of sausage chilled by air cooling (*p* < .05).

### 3.3 Total volatile bases‐nitrogen and thiobarbituric acid reactive substances

**Figure 3:**
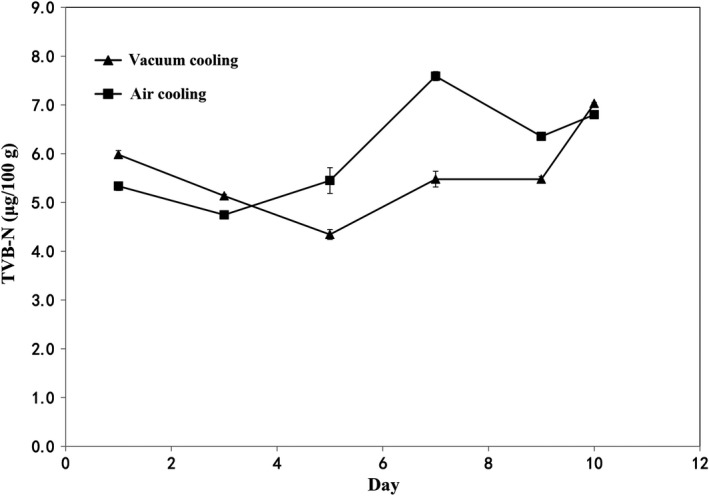
Effects of cooling methods and storage time on total volatile bases‐nitrogen (TVB‐N) of sweet potato sausage stored at 4°C

Total volatile basic nitrogen (TVB‐N) content is one of important index of sausage's freshness (Cai, Chen, Wan, & Zhao, 2011). The results indicated that the trends of TVB‐N values of two cooling methods were generally similar across the time period (Figure 3). Significant differences were still not detected for the TVB‐N values during storage (*p* > .05). Ammonia, trimethylamine, and dimethylamine build up TVB‐N, which originated from the breakdown of nucleotides and from the deamination of amino acids by microorganisms (Campagnoli de Oliveira Filho, Favaro‐Trindade, Trindade, Carvalho Balieiro, & Macedo Viegas, 2010). Therefore, higher TVB‐N values may be caused with higher percentage of protein in the sausage. The protein in sweet potato sausage is generated mainly from pork and egg coating. Therefore, the changes of TVB‐N values during the storage could due to the accumulation of nitrogen compounds, which is produced by the effect of proteolysis.

**Figure 4:**
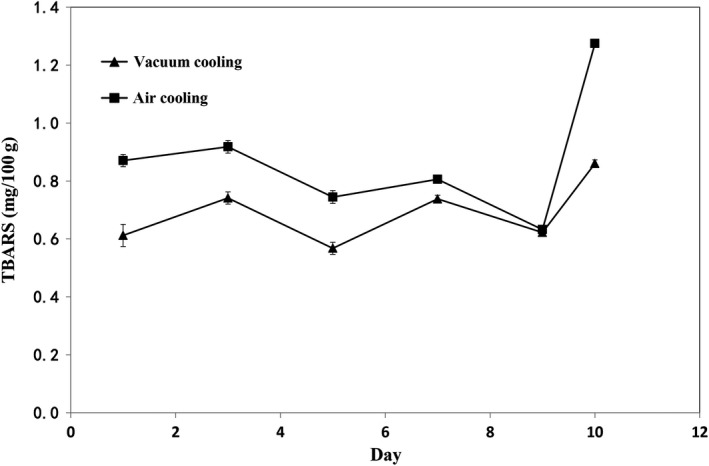
Effects of cooling methods and storage time on thiobarbituric acid reactive substances (TBARS) of sweet potato sausage stored at 4°C

TBARS content, which reflects the degree of lipid oxidation, is widely used as an indicator to determine consumers' acceptability of sausages. The effect of cooling methods was significant (*p* < .05) for TBARS values (Figure 4), while the effect of storage time has no significant difference on the TBARS values (*p *> .05). In the air cooling group, TBARS values slightly changed until the 9th day of storage and then increased exponentially. Sausages with air cooling have higher TBARS content than that of sausages cooled by vacuum cooling. This phenomenon implies that sausages chilled by vacuum cooling had slower growth of reactive substances such as lipid oxidation. Previous studies observed that the TBARS values of sausages increased during refrigerated storage probably due to the increased oxidation caused by unsaturated fatty acids and dehydration (Mendes, Pestana, & Gonçalves, 2008; Zhang, Lin, Leng, Huang, & Zhou, 2013). The oxidative rancidity is recognized as the major cause of reduced quality of sausage (Wenjiao, Yongkui, Yunchuan, Junxiu, & Yuwen, 2014), and this reaction can result in off flavors and color alteration (Coutinho de Oliveira et al., 2012). Therefore, cooked sausages chilled by vacuum cooling have higher quality than that of sausages chilled by air cooling according to the TBARS values.

### 3.4 Moisture content and water activity

**Figure 5:**
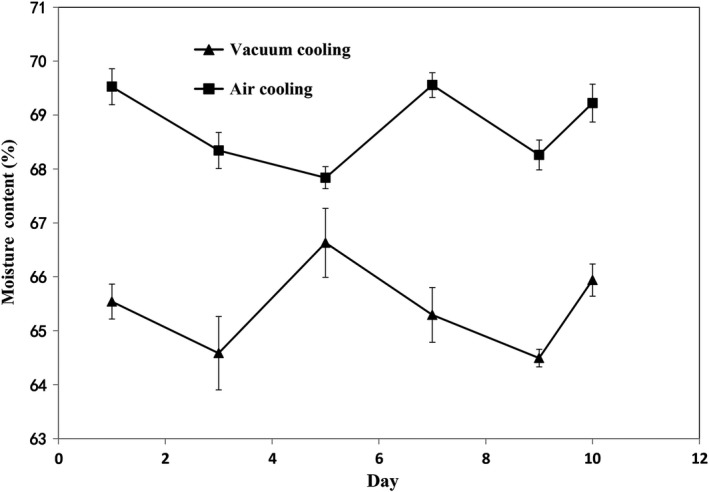
Effects of cooling methods and storage time on moisture content of sweet potato sausage stored at 4°C

Water content is considered to be one of the most influential forces in food chemistry. The results of water content analysis (Figure 5) showed that there were significant differences between two types of cooling methods (*p* < .05). Such water difference can be explained by vacuum cooling caused a large amount of water evaporation, which results in lower water count than that obtained with air cooling. Water content gradually decreased during initial period probably due to dehydration (Feng et al., 2013). The increase of water content after 5th day could be due to starch present in sausages, which can retain moisture in processed meats by hydrophilic natural (Zamudioflores et al., 2015).

**Figure 6:**
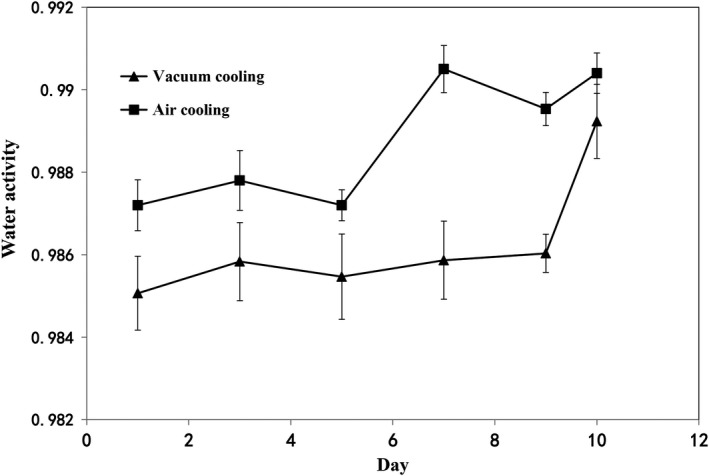
Effects of cooling methods and storage time on water activity of sweet potato sausage stored at 4°C

Water activity (aW) is recognized as a method to find how water influences the chemical reactions in products. Chemical reactions are not only controlled by aW, the growth of microorganisms for spoilage is also influenced by aW. The higher aW in the sausage is the more rapid microorganism grows. The result (Figure 6) shows the effect of cooling methods and storage time on aW of cooked sweet potato sausages stored at 4°C was significant (*p* < .05), but the interaction between the cooling methods and storage time has no significant influence on the aW (*p > *.05). The increased changes in water activity values during storage can be attributed to sausages attaining equilibrium according to the relative humidity of their surroundings. This could explain why sausages gain moisture from cold room (4°C). However, the quality of sausages would be weakened because of the new moisture equilibration with the surrounding humidity. In summary, the difference of water activity (*p* < .05) between vacuum cooling and air cooling probably is due to water evaporation during sample's cooling period.

### 3.5 Instrumental texture measurement

The texture indexes, such as hardness, resilience, springiness, and chewiness, have important impact on consumer acceptability. The results showed significant differences (*p* < .05) between two cooling methods in variables such as hardness, chewiness, and resilience during the evaluation period (Table 5). There were no significant differences in any textural properties among any of the treatments before storage. The hardness of the sausages chilled by air cooling increased significantly (*p* < .05) from day 1, while there were no significant changes for the sausages chilled by vacuum cooling before 5th day of storage. Previous researches have demonstrated the hardness increasement of meat products during refrigerated storage (Estévez, Ventanas, & Cava, 2005, 2006). This might be due to either the water and fat separation from protein matrix caused emulsion destabilization or protein oxidation by developing carbonyls. We speculate that the starch molecules that have gelatinized may undergo retrogradation, resulting in sausages texture firmer. The cooling rate could affect starch retrogradation, which can be described as a process in which starch molecules from disorder to order. As the cooling rate increases, starch retrogradation takes places in a very short period of time and starch molecules have no time to change from a disorderly state to an orderly one, which results in the degree of retrogradation to decrease (Meng, 2007). These findings are agreement with the results of Yu, Ma, Liu, Lucile, and Sun (2010), who reported that higher cooling rates could decrease starch retrogradation at the beginning, but did not retard the changes during the storage. Besides, Campos, Narine, and Marangoni (2002) showed that cooling rates have an effect on the hardness of a fat crystal network, using lard as model system. They found that slow cooling rates results in a softer fat than cooling rapidly and fat became softer with time. However, it was not observed in this work. This phenomenon is probably due to high starch content in sweet potato sausage; thus, the effect of starch retrogradation greater than that of fat crystal network. The hardness of sausages chilled by vacuum cooling gradually became higher than those of sausages chilled by air cooling after 9 days. This is probably be due to the starch retrogradation had been finished after 9 days and sausages chilled by vacuum cooling have lower moisture content than air‐cooled sausages.

**Table 5:** Effects of cooling methods and storage time on instrumental texture of sweet potato sausage stored at 4°C

	Day0	Day1	Day3	Day5	Day7	Day9	Day10
Hardness (g)							
VC	1,306.04 ± 66.23^Aa^	1,389.56 ± 123.70^Aa^	1,515.17 ± 146.78^Aa^	1,941.11 ± 215.07^Ab^	2,756.97 ± 159.34^Ac^	2,904.87 ± 225.79^Ac^	2,892.16 ± 81.05^Ac^
AC	1,272.58 ± 80.83^Aa^	1,758.60 ± 172.59^Bb^	1,999.65 ± 245.15^Bb^	2,423.88 ± 247.27^Bc^	2,839.67 ± 193.52^Ae^	2,573.23 ± 118.29^Bcd^	2,707.40 ± 210.24^Bde^
Springiness (%)							
VC	1.01 ± 0.02^Aa^	0.94 ± 0.04^Ab^	0.88 ± 0.06^Ab^	0.92 ± 0.05^Ab^	0.91 ± 0.03^Ab^	0.92 ± 0.05^Ab^	0.88 ± 0.03^Ab^
AC	0.98 ± 0.04^Aa^	0.85 ± 0.01^Bcd^	0.83 ± 0.05^Acde^	0.91 ± 0.02^Ab^	0.88 ± 0.02^Abc^	0.79 ± 0.05^Be^	0.82 ± 0.06^Ade^
Cohesiveness (%)							
VC	0.77 ± 0.01^Aa^	0.45 ± 0.04^Ab^	0.29 ± 0.07^Ac^	0.25 ± 0.03^Acd^	0.27 ± 0.01^Acd^	0.23 ± 0.03^Ad^	0.24 ± 0.03^Acd^
AC	0.79 ± 0.02^Aa^	0.41 ± 0.04^Ab^	0.23 ± 0.04^Acd^	0.26 ± 0.03^Ac^	0.23 ± 0.02^Bcd^	0.20 ± 0.02^Ad^	0.23 ± 0.02^Acd^
Chewiness							
VC	1,017.21 ± 46.23^Aa^	589.84 ± 90.01^Ab^	379.90 ± 89.42^Ac^	438.32 ± 67.73^Ac^	663.85 ± 10.47^Ab^	602.14 ± 64.48^Ab^	612.05 ± 71.85^Ab^
AC	982.44 ± 50.71^Aa^	619.48 ± 80.24^Ab^	385.5 ± 87.35^Ad^	564.25 ± 86.07^Bbc^	570.89 ± 55.38^Bbc^	400.40 ± 56.13^Bd^	508.07 ± 61.19^Ac^
Resilience (%)							
VC	0.54 ± 0.01^Aa^	0.26 ± 0.03^Ab^	0.18 ± 0.04^Ac^	0.16 ± 0.03^Ac^	0.16 ± 0.01^Ac^	0.14 ± 0.01^Ac^	0.15 ± 0.02^Ac^
AC	0.56 ± 0.02^Aa^	0.23 ± 0.03^Ab^	0.13 ± 0.02^Bcd^	0.15 ± 0.03^Ac^	0.13 ± 0.01^Bcd^	0.11 ± 0.01^Bd^	0.13 ± 0.01^Acd^

Note

The same row with different superscript lowercase letters are significantly different for different storage time (*p* < .05) and different superscript uppercase letters are significantly different for different cooling methods (*p* < .05).

No significant differences (*p* > .05) were detected in the categories of resilience, springiness, chewiness, and cohesiveness between two kinds of cooling methods during the measurements taken at 5th day. However, these values except for chewiness dropped significantly (*p* < .05) as the storage time increased. Chewiness values dropped at initial and then increased significantly (*p* < .05) after 7 days. This phenomenon could be explained by biochemical processes during the storage (Jiménezcolmenero et al., 2010). Above results suggested that vacuum cooling treatments could minimize texture deterioration of sausage during stored at cold room.

## 4 CONCLUSIONS

According to the present results, the shelf life of macro‐porous sausage chilled by vacuum cooling (8 days), with rapid cooling rates and higher moisture loss, is similar to the sausage (9 days) chilled by air cooling. However, cooked sausage cooled by vacuum cooling effectively retarded lipid and protein oxidation as indicated by lower TBARS values and lower contents of TVB‐N. In addition, vacuum‐cooled sausage maintained its texture characteristics for a longer time compared to the air‐cooled sausage. Therefore, the sausage cooled by vacuum cooling has better physicochemical and textural properties than air‐cooled sausage. Thus, applying the vacuum cooling for cooling micro‐porous sausage is useful and may be a practical application for other micro‐porous foods.

## CONFLICT OF INTERESTS

No conflict of interest was declared by the authors.

## ETHICAL APPROVAL

This study does not involve any human or animal testing.
